# Nutritional and Metabolic Consequences of Camelina Seed Oil Compared to Flaxseed Oil in a Rat Diet

**DOI:** 10.3390/molecules30132738

**Published:** 2025-06-25

**Authors:** Reshma Susan Babu, Adam Jurgoński

**Affiliations:** Biological Function of Food Team, Institute of Animal Reproduction and Food Research, Polish Academy of Sciences, Trylińskiego 18 Str., 10-683 Olsztyn, Poland; r.babu@pan.olsztyn.pl

**Keywords:** antioxidant status, lipid metabolism, α-linolenic acid, erucic acid, oxidative stress, palm oil

## Abstract

Camelina seeds are rich in α-linolenic acid (ALA), but also contain small amounts of erucic acid, which is considered toxic to laboratory rats. This experiment compares the dietary inclusion of camelina oil to that of flaxseed oil, a well-known source of ALA, and evaluates their effects on the nutritional and metabolic status of growing rats. The oils were chemically analyzed and incorporated into a semi-purified diet for 4 weeks. The experiment was divided into 3 groups: PO (control-fed palm oil with a trace of ALA), FO (comparative-fed flaxseed oil), and CO (experimental-fed camelina seed oil). Both CO and FO showed a higher percentage of lean body mass, greater lean mass gain, and a lower fat percentage compared to PO. Similar to the body composition, the blood lipid profile also improved in CO and FO, with higher HDL cholesterol and lower triglyceride levels, which was associated with upregulation of the peroxisome proliferator-activated receptor *γ* gene. However, in FO and CO, higher plasma liver enzyme activity and malondialdehyde concentrations were observed in the heart and liver. The results suggest that camelina oil has a similarly beneficial impact on the metabolic processes of the growing body as flaxseed oil, while also indicating a potential for increased organ-specific lipid peroxidation and hepatic burden when consumed in excess.

## 1. Introduction

Camelina (*Camelina sativa* (L.) Crantz) is an ancient oilseed plant of the Brassicaceae family, sometimes called false flax, among other names [1]. This plant is native to temperate Northern Europe and Central Asia and was also introduced to North America as a contaminant in flaxseed [2]. Camelina seeds can comprise up to 43% oil in the dry matter depending on the variety, genotype diversity, climatic and growing conditions, etc. [3]. The oil is usually cold pressed, which makes it rich in fatty acids and bioactive compounds [4]. From a nutritional point of view, camelina oil is highly unsaturated, having polyunsaturated fatty acids (PUFAs) that amount to about 53%. It is especially rich in α-linolenic acid (18:3 n−3) (ALA), approx. 36%, which is found in much lower amounts in common vegetable oils, such as soya oil, sunflower oil, and rapeseed oil [4]. Camelina oil also contains phenolic and sterol compounds like tocopherols and β-sitosterol [4,5]. It possesses a relatively good oxidation resistance, as confirmed by laboratory experiments and long-term oil storage [1].

Flaxseed oil is widely recognized and studied for its beneficial effects, including antioxidant, anti-inflammatory, antibacterial, and anticancer effects [6]. It is high in PUFAs, mainly consisting of about 53% of ALA on average [7]. It is also rich in antioxidants like tocopherols [6], β-carotene [8], and phytosterols that offer substantial metabolic health benefits. Both clinical and preclinical studies underscore flaxseed oil’s ability to reduce the risk of hypertension [9], decrease insulin resistance [10], and increase plasma adiponectin levels [11]. Its lipid-lowering effect relies on lowering plasma total cholesterol, LDL cholesterol, and triglycerides [12] while favorably adjusting the n-6/n-3 ratio in the diet [10].

While both oils are an exceptionally rich source of ALA, camelina oil also contains some amounts of erucic acid (22:1 n-9; up to 4%) [3], a monounsaturated fatty acid considered an antinutrient and toxic compound, especially to the heart [13]. However, the adverse cardiac effect has not been proven in human subjects, whereas in rats, it is reversible [14]. In the European Union, a tolerable daily intake of 7 mg/kg body weight per day and a maximum level of 5% in vegetable oils and fats for erucic acid was established by the European Food Safety Authority (EFSA) [15]. The United States Food and Drug Administration has not determined the safety levels of erucic acid. However, it has a history of banning oils rich in this fatty acid for human consumption, as in the case of mustard oil, which may contain up to 40% erucic acid [16,17]. Erucic acid is efficiently absorbed from the gastrointestinal tract and accumulates in all organs except the brain, though its mitochondrial β-oxidation remains limited in rats. As a consequence of retention, we observe increased mass, decreased feed intake, and impaired function of internal organs like the liver, heart, and kidneys. In rats, this buildup can lead to organ-related disorders like altered lipid composition, hepatocyte vacuolation, kidney tubular dilatation, lipidosis, etc. [15]. However, these changes only occur at extremely high doses (5.5–12.6 g/kg b.wt. per day). Moreover, the narrative on erucic acid has constantly been changing over the past decade, and new data suggest even its beneficial effects on health in reasonable amounts, especially for treating neurodegenerative diseases [14].

From a nutritional perspective, ALA and other n-3 fatty acids are usually deficient in the diet, which results, among other things, from their relatively low content in commonly used vegetable oils [18,19]. For this reason, it is imperative to consider additional sources of these acids, which could diversify the diet and improve the nutritional status of the body in this respect. Even though camelina seed oil contains erucic acid, it has gained attention as a good alternative source of ALA. Considering the modern nutritional trends, it can serve as an additional source of ALA, for example, alongside flaxseed oil, which is a more common and well-investigated source of this nutrient. Interestingly, a recent meta-analysis of clinical trials showed that ingesting camelina oil can improve blood lipid profiles, but only if taken in limited amounts [20]. Thus, this study aimed to understand more about camelina seed oil’s nutritional and health potential by comparing it with flaxseed oil. We hypothesized that due to the high content of ALA, camelina seed oil could positively affect the nutritional and metabolic status of growing rats, which may, however, be limited to some extent by the presence of erucic acid. This research delved into understanding internal organ function, lipid metabolism, and antioxidant status in the growing laboratory rats because ALA is crucial for this period of life.

## 2. Results

### 2.1. Chemical Composition Differences

The saturated fatty acid levels in flaxseed oil (FO) and camelina seed oil (CO) were similar, at 9.51% and 9.14%, respectively. CO has a higher concentration of monounsaturated fatty acids (MUFAs) at 29.5% compared to FO at 18.7%. This was due to the high presence of gondoic acid in CO, which was very low in FO. Erucic acid was only found in CO (2.83%). FO has a higher percentage of PUFAs than CO; both were rich in n-3 PUFAs, exclusively or almost exclusively ALA. It was higher in FO (51.9%) compared to CO (35.1%) (Table 1). Regarding n-6 PUFAs, CO has higher levels (19.5%) than FO (14.9%), primarily linoleic acid (LA), accounting for 17.4% of CO versus 14.9% of FO. CO also contained small amounts of many additional fatty acids that were not present in FO, like 11,14-eicosadienoic acid, 11,14,17-eicosatrienoic acid, 13,16-docosadienoic acid, and nervonic acid (Table 1). 

### 2.2. Dietary Intake, Body Composition, and Internal Organ Weights

Dietary FO and CO did not significantly affect the final body weight or dietary intake. The final body weight increased from 146–148 g to 191–198 g, with a mean increase of 47 g per group. However, significant changes were noticed in body composition at the end of the study. The final fat percentage was also increased in all groups, with the most in the palm oil (PO) group and then FO and the least in the CO group (*p* ≤ 0.05). This was associated with the final lean percentage results, where the FO and CO groups had a higher lean percentage (approx. 63% each) and a lean gain of more than 19 g. The PO group had a percentage of 57, the lowest, with a lean gain of only 5.6 g (Table 2).

**Figure 1:**
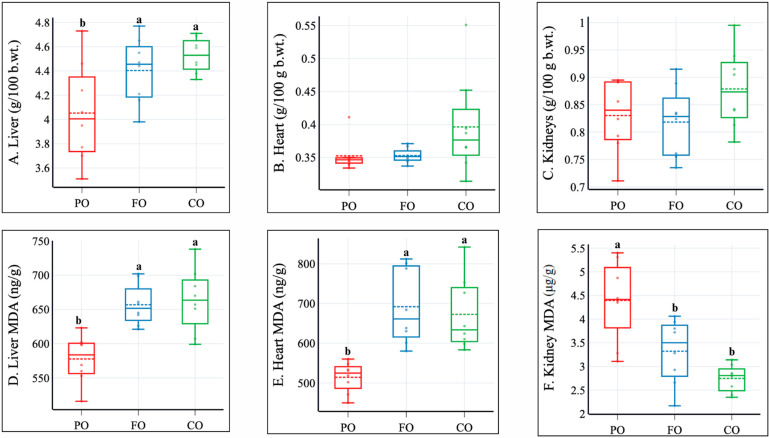
Impact of dietary oils on internal organ weights and malondialdehyde (MDA) concentration. Data are presented as box-and-whisker plots (*n* = 8). PO, control-fed palm oil; FO, comparative-fed flaxseed oil; CO, experimental-fed camelina seed oil. Groups not sharing the same letter above the box plots (a, b) are significantly different (*p* ≤ 0.05) based on Duncan’s or Dunn’s *post hoc* test preceded by one-factor ANOVA or one-factor ANOVA on ranks.

Rats fed with CO and FO demonstrated higher liver weight compared to PO. This can be due to some additional burden of PUFAs on this organ because the dietary presence of camelina and flaxseed oil was high. The liver and heart MDA levels, as markers of lipid peroxidation, are higher in CO and FO compared to PO. Kidney weight had no significant differences across the groups. However, a substantial reduction of MDA in the kidney was observed in FO, particularly in CO, pointing to a contrasting pattern of lipid peroxidation (Figure 1).

### 2.3. Blood Antioxidant Status and Lipid Profile

The plasma markers related to its antioxidant status, including the antioxidant capacity of water-soluble substances (ACW), the antioxidant capacity of lipid-soluble substances (ACL) and uric acid, showed no significant differences across the dietary oil groups (Table 3).

FO and CO benefitted the plasma lipid profile. The HDL cholesterol levels in the FO and CO groups (*p* ≤ 0.05) were higher than in the PO group. The LDL concentrations also significantly differed between the groups, with CO showing the least, followed by FO, and the highest in PO. In contrast, plasma triglycerides were slightly lower in FO than in CO and high in PO (Table 3).

### 2.4. Liver Lipids, Gene Expression, and Plasma Markers of Liver and Kidney Functions

**Figure 2:**
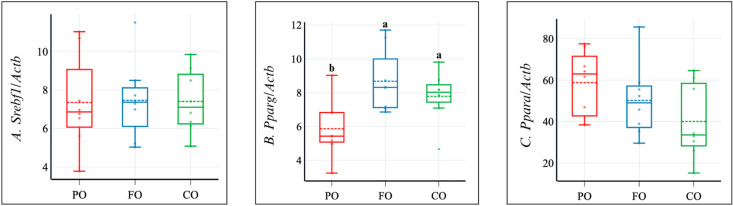
Impact of dietary oils on liver mRNA expression of genes related to lipid metabolism. Data are presented as box-and-whisker plots (*n* = 8). PO, control-fed palm oil; FO, comparative-fed flaxseed oil; CO, experimental-fed camelina seed oil; *Srebf1*, sterol regulatory element-binding protein 1c gene; *Pparg*, peroxisome proliferator-activated receptor gamma gene; *Ppara*, peroxisome proliferator-activated receptor alpha gene; *Actb*, β-actin gene. Groups not sharing the same letter above the box plots (a, b) are significantly different (*p* ≤ 0.05) based on Duncan’s or Dunn’s *post hoc* test preceded by one-factor ANOVA or one-factor ANOVA on ranks.

Liver fat content remained similar among all groups (Table 4). FO and CO showed lower levels of liver triglycerides than PO, but those differences were insignificant (*p* = 0.135), as was the case with cholesterol. The liver expression of sterol regulatory element-binding protein 1c gene (*Srebf1*; *p* = 0.994) and peroxisome proliferator-activated receptor alpha gene (*Ppara*; *p* = 0.115) has no notable changes. In contrast, liver peroxisome proliferator-activated receptor gamma gene (*Pparg*) expression was significantly lower in the PO group than in the other two groups (Figure 2). Furthermore, FO and CO had substantially lower plasma bile acid concentration levels than PO (Table 4).

The inclusion of different dietary oils significantly influenced the liver function in rats. The plasma ALT levels were slightly but notably higher in CO compared to PO. In turn, the plasma ALP levels were higher in FO and CO than in PO. The plasma concentration of other liver and/or kidney function markers, including total bilirubin, creatinine, and urea, remained essentially unchanged (Table 4).

## 3. Discussion

Dietary fats rich in unsaturated fatty acids, specifically PUFAs and MUFAs, are essential and nutritionally beneficial for the body’s basic functions. They serve as the primary energy source and contribute to the cells’ structural and functional integrity, improving the body’s physical functioning and metabolism [21,22]. Vegetable oils are a good source of unsaturated fatty acids, which can serve as bioactive compounds that regulate inflammation, cell signaling, and lipid metabolism [23]. Flaxseed oil is one of the richest and most popular sources of ALA [6]. Camelina oil can be a good additional source, containing more than 30% ALA and 15% LA, which indicates a favorable ALA to LA ratio [24]. Palm oil, used as the control for the experiment, is rich in palmitic acid and thus has some unhealthy effects, suggesting atherogenesis, inflammation, and oxidative stress [25]. 

This experiment explored the metabolic effects of CO, comparing it to FO and PO, highlighting the potential of CO to enhance metabolic health in rats. This model experimental approach focused on the oil source, in which one chosen oil was the only source of fat in the diet. CO and FO are characterized by their high ALA and moderate LA contents compared to most vegetable oils. CO was more varied in fatty acids than FO; however, they were present in relatively small amounts (Table 1). The findings indicate that CO and FO positively influence body composition and lipid metabolism. However, some adverse effects should be considered carefully, including increased lipid peroxidation in the heart and liver (MDA) and plasma markers of liver function.

In this experiment, the overall body composition improved, and the final body fat results based on CO and FO were reduced with an increase in lean mass (Table 2). These changes suggest that the effects driven by both FO and CO helped in lipid metabolism, energy partitioning, and muscle development. PUFAs have been shown to enhance fat metabolism and increase insulin sensitivity [26]. Studies have shown that plant-based oils rich in PUFAs have reduced body fat and improved lean mass, specifically with ALA and LA [27]. As PO contained LA (9%) and only traces of ALA (0.16%), our results emphasize the crucial importance of ALA in developing favorable body composition during animal growth.

Improvements in HDL cholesterol levels were primarily attributed to the effects of FO and CO in this experiment. PO was seen with increased LDL cholesterol levels due to the high levels of saturated fatty acids (SFAs) in this oil (Table 3) [25]. FO is known to improve HDL cholesterol and reduce LDL cholesterol, mainly due to the positive effects of n-3 fatty acids [12]. CO, with its higher MUFA proportion and the presence of erucic acid comparatively, also decreased plasma triglycerides and increased HDL cholesterol. Evidence from human trials supports CO’s cholesterol-lowering effects in the blood, which were observed over a longer period (>8 weeks) and only at limited doses (<30 g/day). Still, its impact on the blood triglycerides remained negligible [20]. Enhanced HDL levels correlate with cholesterol reversal and possess antithrombotic, antioxidant, and anti-inflammatory effects [28]. However, the plasma antioxidant status did not differ between groups in this experiment. In turn, the improvement of blood lipids can be associated with the significant upregulation of *Pparg* in FO and CO, especially by ALA. It is known that ALA can activate *Ppars*, including *Pparg*, which promotes lipid storage [29], and *Ppara,* which promotes β-oxidation of fatty acids [30]. Increased *Pparg* expression was also seen in diabetic rats in a CO and high-intensity interval training supplementation study [31]. Higher *Pparg* expression is linked with improved plasma HDL cholesterol and triglyceride levels, and the latter effect is caused by an increase in lipoprotein lipase-dependent triglyceride clearance [32]. Typically, oils containing erucic acid, like erucic acid-rich rapeseed oil, have been associated with adverse metabolic effects, including increased triglyceride, total, and LDL cholesterol levels and decreased body weight [33]. The decline in plasma LDL cholesterol and triglycerides with increased plasma HDL cholesterol by CO (Table 3) implies that erucic acid in CO did not negatively affect the blood lipid profile. Apparently, these positive effects were predominantly driven by the oil’s PUFAs.

The high levels of PUFAs in vegetable oils can induce lipid peroxidation, forming peroxides and reactive oxygen species. Theoretically, they are more prone to oxidation than SFAs and MUFAs in the body, and the presence of two or more double bonds enables the formation of conjugated dienes during oxidation [34]. The peroxidation of PUFAs, such as ALA, arachidonic acid, and others, produces MDA; thus, it is a key biomarker of oxidative stress and potential cellular damage [35]. PUFAs require antioxidants to prevent peroxidation [36], which can further control the MDA levels. PUFA-rich oils exhibit variable effects on the antioxidant status of the body depending on their source and the experimental design. To the best of our knowledge, this is the first report of an increase in lipid peroxidation induced by camelina oil and flaxseed oil compared to palm oil. However, similar results have been reported in the literature, where linoleic acid-rich corn oil and docosahexaenoic acid-rich fish oil increased lipid peroxidation in healthy rats compared to more saturated sources of fat, such as butter, margarine, and coconut oil [35,37]. Interestingly, supplementation of postmenopausal women with fish oil rich in n-3 PUFAs was not associated with greater in vivo lipid peroxidation compared to oils rich in oleate and linoleate [38]. However, in another study on diabetic rats, antioxidant activities were even attributed to flaxseed oil when corn oil was used as a control [39].

Despite higher MUFA content in CO than FO, which generally are more stable fatty acids [34], we observed similar MDA results in both the comparative and experimental groups. This outcome may somehow be associated with CO’s erucic acid presence and its tendency to over-accumulate in internal organs [40]. CO contained 2.83% erucic acid, which, upon calculations, gives an exposure of 139 mg/kg b.wt. per day. This is 20 times higher than the tolerable daily intake (TDI) for humans established by EFSA (7 mg/kg/b.wt./d) [15]. Nevertheless, other components of the cold-pressed oils, especially tocopherols, might have also played a role [8]. Interestingly, the kidney MDA levels were lower in FO and CO, suggesting the organ-specific response to the oil’s components. In addition, renal functions appear to be unaffected by CO and FO (Table 4). These findings, in association with the stable antioxidant status of blood plasma (Table 3), suggest the oils induced no systemic oxidative stress but rather an organ-specific one (liver and heart) [41]. Since some liver markers were higher in FO and CO (ALT and ALP, Table 4), the increased lipid peroxidation may have a detrimental impact on their hepatic functions.

Preclinical studies are essential to ensure safety for products with potential health risks, especially when they may also be a good source of nutrients that are typically limited in the diet, like ALA. Currently, human data are insufficient for establishing a reliable dose-response evaluation and are not entirely conclusive. Human exposure to erucic acid, as evidenced by biomonitoring studies, shows variable concentrations in plasma [42]. Some epidemiological data indicate associations with heart failure and cancer risks, but the causality remains rather weak [43,44]. Therapeutic exposure to adrenoleukodystrophy, a rare genetic disorder which was previously treated with erucic acid-rich Lorenzo’s oil, has been linked to hematological changes but not myocardial toxicity [15,45].

## 4. Materials and Methods

### 4.1. Chemical Composition of Camelina Oil

The unrefined, cold-pressed camelina seed oil and flaxseed oil were purchased from Olvita L.P. (Marcinowice, Lower Silesian Voivodeship, Poland), a company specializing in producing cold-pressed vegetable oils. The refined palm oil was purchased at a local supermarket (Metro AG), which has its own brand. The fatty acid profiles of all oils were quantified in triplicate by an accredited testing laboratory (Nuscana, Mrowino, Poland) under the Polish-European ISO standard (PN-EN ISO 12966-1:2015 and 12966-2: 2011), using gas chromatography with flame-ionization detection after previous conversion of the fatty acids into respective methyl esters [46]. The fatty acid profile of flaxseed and camelina seed oils is shown in Table 1, while that of palm oil is from a previously published paper [47] and given in Table 5.

### 4.2. Animals, Diets, and Experimental Design

The feeding experiment was conducted on lean (Fa/?) female Zucker rats allocated to three groups of eight animals each and fed for 4 weeks with a semi-purified diet (Table 5). The rats were approximately 6 weeks old; their initial and final body weight is shown in Table 2. Each group was fed a modified version of the semi-purified casein diet recommended for growing rodents by Reeves [48]. The diets were identical in fat composition, with 7% of oil incorporated, varying solely by the type of oil. The control group was fed a diet containing palm oil (PO group) with a high percentage of SFAs, mainly palmitic acid (45%) and only a trace of ALA (0.16%). Refined palm oil was selected as the control because it is a commonly used source of SFAs and contains only trace amounts of ALA. This choice enabled the assessment of the metabolic effects of the tested oils rich in ALA in growing rats, which are sensitive to nutrient deficiencies, over a relatively short experimental period. The reference group was fed a diet containing flaxseed oil (FO group), and the experimental group was fed a diet containing camelina seed oil (CO group), both rich in ALA. The experiment was model-based and focused specifically on the oil source. Each oil was used as a primary source of fat to observe the differences in their effects on the rat’s body. A detailed description of the diet, which was freely available to the rats, is shown in Table 5. The rats were individually housed in plastic cages in a controlled environment (12-h light/dark cycle, temperature of 21 ± 1 °C, relative humidity of 55 ± 10%, and 15 air changes per hour). The experiment protocol followed European Union legislation for the care and use of laboratory animals (Directive 2010/63/EU) and ARRIVE guidelines and was approved by the Local Institutional Animal Care and Use Committee in Olsztyn, Poland (permission number: 37/2017).

### 4.3. Sampling and Analysis of Biological Material

The rats were anaesthetized at the end of the experimental feeding with a combination of xylazine and ketamine in physiological salt (10 mg and 100 mg/kg body weight, respectively). After weighing each rat, the abdomen was cut open. Blood was drawn from the vena cava into specific heparinized tubes. It was then centrifuged for 10 min at 380× *g* and 4 °C. The resulting plasma was refrigerated until further analysis. Other organs, including the liver, heart, and kidneys, were taken out, weighed, and frozen in liquid nitrogen or used for further analysis.

Liver lipids were obtained with the Folch method [49] with previously outlined modifications [50]. In summary, the liver tissue was homogenized with a 2:1 blend of chloroform-methanol in a homogenizer (IKA T25, Wilmington, NC, USA) and then centrifuged at 15,000× *g* for 10 min. The supernatant was rinsed with distilled water, vortexed, and centrifuged for 15 min at 2500× *g*. Once the upper phase is removed, the lower phase with lipids is evaporated using a nitrogen stream at 37 °C. The lipid fraction seen in this manner was subsequently dissolved in chloroform. Then, the liver’s fat, triglycerides, and cholesterol concentrations were measured spectrophotometrically in this solution with reagents provided by Alpha Diagnostics Ltd. (Warsaw, Poland). 

The malondialdehyde (MDA) concentration in the liver, kidney, and heart was measured spectrophotometrically (at 532 nm) using the extraction method described by Botsoglou et al. [51]. It was given as ng and µg of MDA per gram of organ.

The levels of cholesterol in blood plasma, i.e., total, HDL and LDL, and triglycerides, along with the plasma activity of alanine transaminase (ALT), aspartate transaminase (AST), and alkaline phosphatase (ALP), were determined using a biochemical analyzer (Pentra C200, Horiba Ltd., Kyoto, Japan). Additionally, urea, creatinine, uric acid, and total bilirubin were assessed. The total concentration of bile acids in the blood plasma was determined using the Cell Biolabs kit (San Diego, CA, USA). The antioxidant capacity of water- and lipid-soluble substances, i.e., ACW µg/mL and ACL µg/mL, was determined using Photochem (Analytik Jena AG, Jena, Germany). The method relies on producing free radicals, which are then partially removed with antioxidants found in the plasma sample. Then, the concentration of free radicals generated is measured based on luminescence generation. Ascorbate and Trolox are used as standards for ACW and ACL, respectively, in the calibration curves.

### 4.4. mRNA Quantification

The mRNA expression levels of genes in the liver were measured following a previously published method [50], along with equipment and reagents from Thermo Fisher Scientific (Waltham, MA, USA). The total RNA isolation (TRI) reagent solution was used to extract RNA from the liver. The quantity and quality of RNA were measured spectrophotometrically with a NanoDrop1000 device and agarose gel electrophoresis (Thermo Fisher Scientific, Waltham, MA, USA), respectively. A ribonuclease inhibitor and a high-capacity cDNA reverse transcription kit were used to synthesize DNA from 500 ng of RNA. Expression data were normalized to *Actb* and multiplied by 10. Previous studies [50,52] have demonstrated that *Actb* maintains stable expression across comparable experimental settings, supporting its use in this study as the reference gene. The primers used were rat *Srebf1* (ID No.: Rn01495769_m1), rat *Ppara* (ID No.: Rn00566193_m1), rat *Pparg* (ID No.: Rn00440945_m1), and rat *Actb* (ID No.: Rn00667869_m1). Single Tube TaqManVR Gene Expression Assays were used to quantify *Ppara, Pparg*, and *Srebf1* mRNA expression levels (Life Technologies, Carlsbad, CA, USA). The 7900HT Fast Real-Time PCR System was used to perform amplification with the following parameters: initial denaturation for 10 min at 95 °C, followed by 40 cycles of 15 s at 95 °C and 1 min at 60 °C. 

### 4.5. Statistical Analysis

The results are expressed as the mean ± standard error of the mean (SEM), except for the chemical composition of FO and CO, the results of which were expressed as the mean ± standard deviation (SD). One-factor analysis of variance (ANOVA) and Duncan’s post hoc test were applied to identify significant group differences at *p* ≤ 0.05. Dunn’s Bonferroni-corrected post hoc test (*p* ≤ 0.05) was conducted after a one-factor Kruskal–Wallis ANOVA by ranks if the ANOVA was not homogeneous. Statistica version 13.1 (StatSoft Corp., Cracow, Poland) was used for all computations.

## 5. Conclusions

The health benefits of consuming cold-pressed camelina seed oil have been indicated here, and they are similar in scope to flaxseed oil obtained in an analogous process. These benefits are generally related to improving body composition and lipid metabolism in growing healthy rats. Our model study suggests that camelina seed oil and flaxseed oil, both rich in ALA, have a beneficial impact on the metabolic processes of the growing body. However, it also indicates a potential increase in organ-specific lipid peroxidation and liver burden when these oils are excessively present in the diet, which is a clear limitation of their wider use. The comparison between oils suggests that the presence of erucic acid does not appear to have negatively contributed to the biological effects of camelina seed oil. However, this has to be verified in further dose-dependent experiments, including those reflecting pathophysiological conditions, and potentially in human trials.

## Figures and Tables

**Table 1:** Fatty acid profile of flaxseed and camelina seed oils ^1^ (%).

Fatty Acid	Flaxseed Oil	Camelina Seed Oil
Palmitic acid (16:0)	5.40 ± 0.01	5.08 ± 0.02
Stearic acid (18:0)	3.89 ± 0.01	2.21 ± 0.01
Oleic acid (18:1 n-9)	17.6 ± 0.01	12.2 ± 0.06
Vaccenic acid (18:1 n-7)	1.03 ± 0.01	1.06 ± 0.01
Erucic acid (22:1 n-9)	–	2.83 ± 0.01
Linoleic acid (18:2 n-6)	14.7 ± 0.02	17.4 ± 0.01
Arachidic acid (20:0)	0.11 ± 0.01	1.37 ± 0.00
γ-Linolenic acid (C18:3 n-6)	0.22 ± 0.00	0.12 ± 0.00
Gondoic acid (20:1 n-9)	0.09 ± 0.00	12.8 ± 0.03
α-Linolenic acid (18:3 n-3) ALA	51.9 ± 0.03	35.1 ± 0.05
11,14-Eicosadienoic acid (20:2 n-6)	–	1.82 ± 0.01
Behenic acid (22:0)	0.11 ± 0.00	0.31 ± 0.00
11,14,17-Eicosatrienoic acid (20:3 n-3)	–	1.36 ± 0.01
13,16-Docosadienoic acid (20:2 n-6)	–	0.18 ± 0.00
Lignoceric acid (24:0)	–	0.17 ± 0.00
Nervonic acid (24:1 n-9)	–	0.62 ± 0.01
Other (unidentified)	0.25 ± 0.02	0.48 ± 0.00
SFAs ^2^	9.51 ± 0.01	9.14 ± 0.02
MUFAs ^2^	18.7 ± 0.01	29.5 ± 0.02
PUFAs ^2^	66.8 ± 0.01	56.0 ± 0.03
n-3	51.8 ± 0.02	36.5 ± 0.03
n-6	14.9 ± 0.01	19.5 ± 0.01

^1^ Values are means ± SDs (*n* = 3). ^2^ SFAs, saturated fatty acids; MUFAs, monounsaturated fatty acids; PUFAs, polyunsaturated fatty acids.

**Table 2:** Impact of dietary oils on body weight, dietary intake, and body composition in rats.

	Group			ANOVA *p* Value
	PO	FO	CO	
Initial body weight (g)	148 ± 2.27	146 ± 2.25	147 ± 1.65	NS
Initial fat (%)	20.5 ± 0.704	19.0 ± 0.733	18.6 ± 0.879	NS
Initial lean (%)	70.0 ± 0.810	72.4 ± 0.943	72.7 ± 1.041	NS
Final body weight (g)	191 ± 4.29	198 ± 4.46	197 ± 3.10	NS
Dietary intake (g/day)	13.7 ± 0.318	13.5 ± 0.252	13.8 ± 0.166	NS
Final fat (%)	32.6 ± 1.46 ^a^	27.4 ± 1.21 ^b^	27.2 ± 1.19 ^b^	<0.05
Final lean (%)	57.1 ± 1.11 ^b^	63.6 ± 1.18 ^a^	63.9 ± 1.32 ^a^	0.001
Body weight gain (g)	43.7 ± 2.80	51.9 ± 2.70	50.1 ± 3.09	NS
Fat gain (g)	32.5 ± 4.11	26.7 ± 3.31	26.3 ± 2.10	NS
Lean gain (g)	5.58 ± 1.88 ^b^	19.87 ± 1.78 ^a^	19.19 ± 2.90 ^a^	<0.001

Values are means ± SEMs (*n* = 8). PO, control-fed palm oil; FO, comparative-fed flaxseed oil; CO, experimental-fed camelina seed oil; NS, non-significant. ^a,b^ *p* ≤ 0.05 if labelled means in a row do not share the same letter (Duncan’s or Dunn’s post hoc test preceded by one-factor ANOVA or one-factor ANOVA on ranks).

**Table 3:** Impact of dietary oils on markers of antioxidant status and lipid profile in the blood plasma.

	Group			ANOVA *p* Value
	PO	FO	CO	
Antioxidant status				
Uric acid (µmol/L)	24.9 ± 6.37	22.5 ± 1.80	22.9 ± 3.83	NS
ACW (µg/mL)	1.51 ± 0.298	1.07 ± 0.107	1.60 ± 0.402	NS
ACL (µg/mL)	11.9 ± 0.860	11.6 ± 0.923	13.2 ± 0.555	NS
Lipid profile				
HDL cholesterol (mmol/L)	0.663 ± 0.037 ^b^	0.834 ± 0.034 ^a^	0.813 ± 0.051 ^a^	<0.05
LDL cholesterol (mmol/L)	0.115 ± 0.008 ^a^	0.098 ± 0.004 ^ab^	0.086 ± 0.006 ^b^	0.01
Cholesterol (mmol/L)	2.21 ± 0.082	2.05 ± 0.094	2.24 ± 0.132	NS
Triglycerides (mmol/L)	3.96 ± 0.606 ^a^	1.82 ± 0.122 ^b^	2.14 ± 0.215 ^ab^	<0.01

Values are means ± SEMs (*n* = 8). PO, control-fed palm oil; FO, comparative-fed flaxseed oil; CO, experimental-fed camelina seed oil; ACW, the antioxidant capacity of water-soluble substances; ACL, the antioxidant capacity of lipid-soluble substances; NS, non-significant. ^a,b^ *p* ≤ 0.05 if labelled means in a row do not share the same letter (Duncan’s or Dunn’s post hoc test preceded by one-factor ANOVA or one-factor ANOVA on ranks).

**Table 4:** Impact of dietary oils on liver lipids and markers of liver and kidney functions in the blood plasma of rats.

	Group			ANOVA *p* Value
	PO	FO	CO	
Liver				
Fat (%)	8.90 ± 0.289	8.62 ± 0.180	8.14 ± 0.518	NS
Triglycerides (mg/g)	6.02 ± 0.566	4.59 ± 0.627	4.66 ± 0.415	NS
Cholesterol (mg/g)	1.25 ± 0.033	1.20 ± 0.079	1.13 ± 0.043	NS
Plasma markers				
ALT (U/L)	25.6 ± 1.21 ^b^	29.9 ± 2.07 ^ab^	32.5 ± 1.45 ^a^	<0.01
AST (U/L)	54.4 ± 3.24	55.6 ± 3.16	54.7 ± 3.11	NS
ALP (U/L)	175 ± 9.21 ^b^	240 ± 22.224 ^a^	265 ± 15.0 ^a^	<0.01
Bile acids (µmol/L)	15.5 ± 0.739 ^a^	11.4 ± 0.209 ^b^	11.3 ± 0.382 ^b^	0.001
Total bilirubin (µmol/L)	3.53 ± 0.725	3.65 ± 0.850	3.23 ± 0.425	NS
Creatinine (µmol/L)	9.40 ± 1.63	10.8 ± 2.19	8.70 ± 1.85	NS
Urea (mmol/L)	6.70 ± 0.257	6.36 ± 0.239	6.72 ± 0.228	NS

Values are means ± SEMs (*n* = 8). PO, control-fed palm oil; FO, comparative-fed flaxseed oil; CO, experimental-fed camelina seed oil; AST, aspartate transaminase; ALT, alanine transaminase; ALP, alkaline phosphatase; MDA, malondialdehyde; NS, non-significant. ^a,b^ *p* ≤ 0.05 if labelled means in a row do not share the same letter (Duncan’s or Dunn’s post hoc test preceded by one-factor ANOVA or one-factor ANOVA on ranks).

**Table 5:** Composition of the diets (g/100 g).

Ingredient (%)	Group ^1^		
	PO	FO	CO
Casein ^2^	20.00	20.00	20.00
DL-methionine	0.3	0.3	0.3
Palm oil ^3^	7	–	–
Flaxseed oil ^4^	–	7	–
Camelina seed oil ^4^	–	–	7
Corn starch	53	53	53
Sucrose	10	10	10
Cellulose	5	5	5
Mineral mix ^5^	3.5	3.5	3.5
Vitamin mix ^5^	1	1	1
Choline chloride	0.2	0.2	0.2

^1^ PO, control-fed palm oil; FO, comparative-fed flaxseed oil; CO, experimental-fed camelina seed oil. ^2^ Casein preparation (g/100 g): crude protein, 88.7; crude fat, 0.3; ash, 2.0; water, 8.0. ^3^ Fatty acid profile: SFAs, 46.3 including palmitic acid, 40.8; MUFAs, 39.4 including oleic acid, 39.1; PUFAs, 9.21 including linoleic acid, 9.1 [47]. ^4^ Chemical composition in Table 1. ^5^ Recommended for the AIN-93G diet.

## Data Availability

Dataset available on request from the authors.

## Abbreviations

The following abbreviations are used in this manuscript:

PUFAs	Polyunsaturated fatty acids
ALA	α-Linolenic acid
PO	Palm oil
FO	Flaxseed oil
CO	Camelina seed oil
MDA	Malondialdehyde
LA	Linoleic acid
LDL	Low-density lipoprotein
HDL	High-density lipoprotein
EFSA	European Food Safety Authority
FDA	Food and Drug Administration
MUFAs	Monounsaturated fatty acids
NS	Non-significant
ACW	Antioxidant capacity of water-soluble substances
ACL	Antioxidant capacity of lipid-soluble substances
*Srebf1*	Sterol regulatory element-binding protein 1c gene
*Pparg*	Peroxisome proliferator-activated receptor gamma gene
*Ppara*	Peroxisome proliferator-activated receptor alpha gene
*Actb*	β-actin gene
AST	Aspartate transaminase
ALT	Alanine transaminase
ALP	Alkaline phosphatase
SFAs	Saturated fatty acids
TDI	Tolerable daily intake
TRI	Total RNA isolation reagent
SEM	Standard error of the mean
SD	Standard deviation
ANOVA	One-factor analysis of variance
